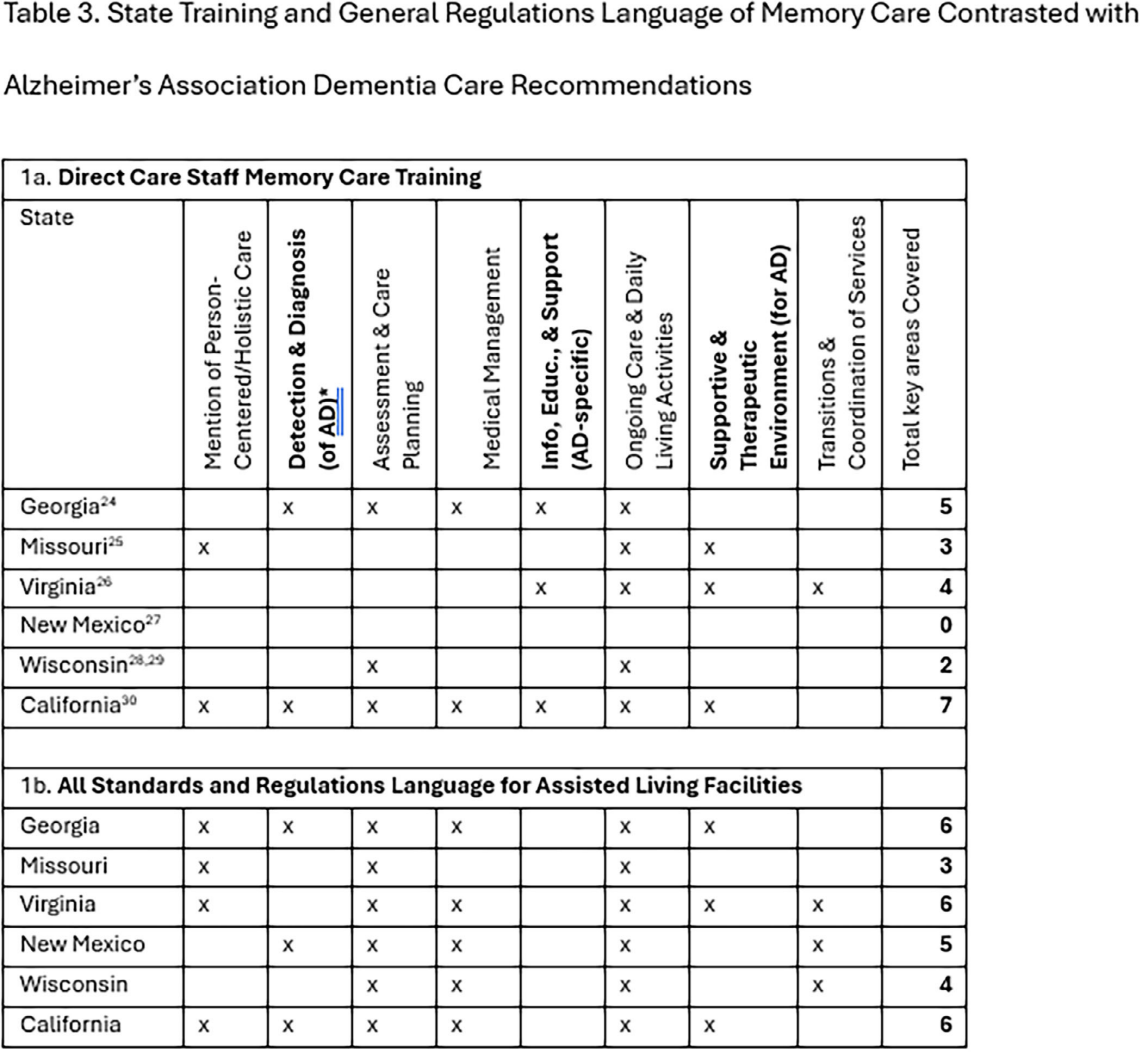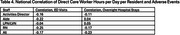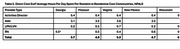# A Snapshot of Memory Care: Regulation, Staffing, and Adverse Events in Memory Care Units within Assisted Living Facilities

**DOI:** 10.1002/alz70860_101141

**Published:** 2025-12-23

**Authors:** Margaret Manhester, Jalayne J. Arias, Victoria Helmly, Catherine E. A. Scipion, Alice Prendergast, Olusheun Olupitan

**Affiliations:** ^1^ Georgia State University School of Public Health, Atlanta, GA, USA; ^2^ School of Public Health ‐ Georgia State University, Atlanta, GA, USA; ^3^ Georgia State University, Atlanta, GA, USA; ^4^ Georgia State University College of Law, Atlanta, GA, USA

## Abstract

**Background:**

Quality of memory care varies across states and can affect health and quality of life outcomes for persons living with dementia (PLWD) within assisted living facilities (ALFs). This study evaluates regulations of memory care units in ALFs across six selected states as potential drivers of health and quality of life outcomes reported within the National Post‐acute and Long‐term Care Study (NPALS).

**Methods:**

Empirical legal methods were used to map state regulations that guide training standards, staff ratios, and direct care staff time spent with residents. Comparative analysis was used to understand relationships between regulations and adverse events.

**Results:**

Within the six‐state snapshot, significant variation of regulations may affect quality of care in memory care centers. Day‐shift direct care staff ratios varied significantly across states (e.g., California: 1:10, Virginia: 4:40; compared to Wisconsin requires only presence of 1 Direct Care Staff). Yet, in Wisconsin, residents must receive 3 hours of individual nursing care per week and average hours per day/resident is 6.1 hours ‐ highest of all six states. Comparatively, average hours per resident/day were lowest in New Mexico (4.7 hours) and falls below the national average (4.9). California also falls below the national average (3.9). States requirements in care and daily living activities were consistent with Alzheimer's Association Memory Care Recommendations, except for New Mexico. Although, states with training requirements that are consistent with the Alzheimer's Association recommendations may not follow all eight of the recommendations. For example, California state regulations meet 7 of 8 the Alzheimer's Association recommendations. The findings from this study indicate that inclusion of standardized memory care training and greater direct care time with residents in ALF policy may enhance care quality. Standardization and inclusion of memory care curriculum and greater direct care time with residents also may enhance care quality.

**Conclusion:**

The results indicate that states with policy language that is more aligned with the Alzheimer's Association 2018 Dementia Care Recommendations may drive lower hospital visit rates, higher direct care staff time spent with residents, while staff ratio requirements may not. Aide and RN hours spent with residents may also influence the likelihood of resident ED visits.